# Protocol and baseline data from The Inala Chronic Disease Management Service evaluation study: a health services intervention study for diabetes care

**DOI:** 10.1186/1472-6963-10-134

**Published:** 2010-05-24

**Authors:** Deborah A Askew, Claire L Jackson, Robert S Ware, Anthony Russell

**Affiliations:** 1 Discipline of General Practice, School of Medicine, The University of Queensland, Brisbane, Queensland, Australia; 2 School of Population Health, The University of Queensland, Brisbane, Queensland, Australia; 3 Diamantina Institute for Cancer, Immunology and Metabolic Medicine, The University of Queensland, Brisbane, Queensland, Australia; 4 Department of Diabetes and Endocrinology, Princess Alexandra Hospital, Woolloongabba, Queensland, Australia

## Abstract

**Background:**

Type 2 Diabetes Mellitus is one of the most disabling chronic conditions worldwide, resulting in significant human, social and economic costs and placing huge demands on health care systems. The Inala Chronic Disease Management Service aims to improve the efficiency and effectiveness of care for patients with type 2 diabetes who have been referred by their general practitioner to a specialist diabetes outpatient clinic. Care is provided by a multidisciplinary, integrated team consisting of an endocrinologist, diabetes nurse educators, General Practitioner Clinical Fellows (general practitioners who have undertaken focussed post-graduate training in complex diabetes care), and allied health personnel (a dietitian, podiatrist and psychologist).

**Methods/Design:**

Using a geographical control, this evaluation study tests the impact of this model of diabetes care provided by the service on patient outcomes compared to usual care provided at the specialist diabetes outpatient clinic. Data collection at baseline, 6 and 12-months will compare the primary outcome (glycaemic control) and secondary outcomes (serum lipid profile, blood pressure, physical activity, smoking status, quality of life, diabetes self-efficacy and cost-effectiveness).

**Discussion:**

This model of diabetes care combines the patient focus and holistic care valued by the primary care sector with the specialised knowledge and skills of hospital diabetes care. Our study will provide empirical evidence about the clinical effectiveness of this model of care.

**Trial registration:**

Australian New Zealand Clinical Trials Registry ACTRN12608000010392.

## Background

Type 2 Diabetes Mellitus (T2DM) is one of the most disabling chronic conditions worldwide, resulting in significant human, social and economic costs and placing huge demands on health care systems[1]. It affects more than 880,000 Australians 25 years or older[2], and the prevalence is increasing as more people develop the condition, the detection of the condition improves, and people with the condition live longer[3]. People with T2DM are at risk of acute and chronic micro- and macro-vascular complications including retinopathy, nephropathy, neuropathy, peripheral vascular disease, coronary heart disease and stroke; as well as mental health problems associated with living with a chronic disease. The United Kingdom Prospective Diabetes Study has demonstrated that rigorous glycaemic control can significantly reduce diabetic complications[4], underscoring the need for, and benefits of, early diagnosis and appropriate management.

In Australia, most people with T2DM receive the majority of their diabetes care from their general practitioner (GP)[5]. In 2006/07, it was the second most frequently managed chronic problem in Australian general practice, and was the reason for 3.7% of general practice encounters; a 42% increase from 1998/99[6]. Around 5% of consultations for diabetes lead to a referral by the GP for specialist level care, most commonly to specialist diabetes outpatient clinics[6]. However, due to the increasing prevalence of the condition and the finite capacity of specialist outpatient clinics, new models of meeting community needs for complex diabetes management are required.

The Chronic Care Model (CCM) is a well known model of care for people with chronic conditions that suggests that optimal health outcomes are achieved when a prepared and proactive practice team interacts with informed and activated patients[7]. This model has informed policy and been adapted for use in different countries and different health care systems for caring for patients with various chronic conditions, including T2DM[8]. The model describes the six elements considered to be essential for improving the care of people with chronic disease. These elements include delivery system design, self management support, decision support, clinical information systems, community resources and health care organisations.

The Inala Chronic Disease Management Service (ICDMS) is a new model of T2DM care that is informed by the CCM with a particular focus on redesigning the health care delivery system and improving patients' self management skills - two of the six key elements of the CCM. The conceptualisation of the ICDMS was informed by the knowledge that there were unacceptably long waiting lists for patients to gain access to specialist diabetes outpatient clinics; the belief that with adequate training and support, primary care providers could provide high quality care for people with diabetes; and the desire to increase efficiency of care by making better use of the skills of providers. The aim of the intervention was to improve the efficiency and effectiveness of care with the flow on effects of potentially being more cost effective. This paper describes the ICDMS clinical model and the methods used to evaluate it, and provides baseline data from the participants. The discussion highlights the challenges faced, and the trade-offs between service delivery and experimental control in the context of this effectiveness trial.

### The ICDMS clinical model

**Figure 1:**
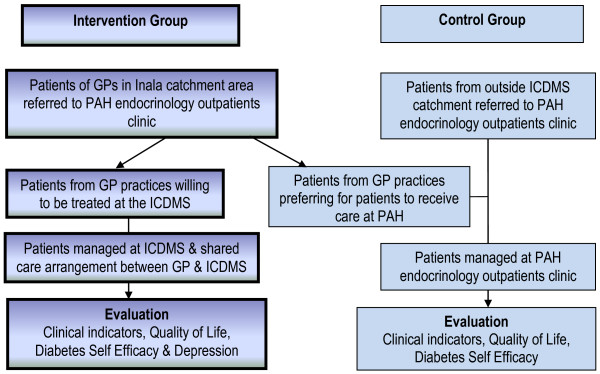
**Flowchart describing study design for evaluating Inala Chronic Disease Management Service clinical model**.

The ICDMS operates from within a general practice that is co-located within the Inala Community Health Centre. Care is provided by a multidisciplinary team consisting of an endocrinologist, GP Clinical Fellows (GPs who have undertaken focussed post-graduate training in complex diabetes care), diabetes nurse educators, dietitian, podiatrist and psychologist. The ICDMS clinical model has three discrete components: the assessment and complications screening; the weekly multidisciplinary diabetes clinic; and the review and discharge phase. The ICDMS clinical model aims to enable each patient achieve target levels for blood glucose, lipids, and blood pressure (BP), or as close to target as realistic, and to then return to their referring GP as soon as practicable for their ongoing diabetes care. The key elements of the clinical model are presented in Figure 1.

### Comprehensive assessment and complications screening

At the patient's first visit to the ICDMS, a diabetes nurse educator completes a comprehensive assessment and complications screening. This assessment includes anthropometric data, self-reported current medications (prescription, over-the-counter medications, and complementary medicines), relevant diabetes history (year of diagnosis, family history of diabetes, previous gestational diabetes, frequency of hypoglycaemic episodes, day-to-day glycaemic control, and frequency of self monitoring of blood glucose levels), smoking status, alcohol consumption, current physical activity levels, weight management, and relevant medical history including known macro- and microvascular complications of diabetes. Retinopathy screening is conducted on site using a non-mydriatric camera with retinal photographs taken by a trained nurse and interpreted by a trained GP[9]. The patient's feet are screened, and any patients with acute foot complications or with a "high risk" foot are immediately referred to the ICDMS podiatrist for assessment. Results from existing pathology tests for glycated haemoglobin (HbA1c), serum lipids, folate, vitamin B12, creatinine, liver function tests, and urinary albumin:creatinine ratio are used if tested within the previous three months. If not, patients are provided with pathology request slips. An onsite, publicly funded, pathology collection point facilitates the timely processing of requests.

### Weekly multidisciplinary clinic

The ICDMS multidisciplinary clinic is conducted one morning per week and is serviced by all members of the team (endocrinologist, GP Clinical Fellows, diabetes nurse educators, dietitian, podiatrist, and psychologist). In consultation with the patient, the GP Clinical Fellow reviews the information gathered during the patient's assessment and complications screening, determines the patient's own goals for diabetes treatment, and develops a patient specific management plan to address the identified issues. The endocrinologist reviews the management plans and co-consults with the GP Clinical Fellow if the patient's clinical issues are particularly complex. Evidence-based guidelines or protocols inform management of hyperglycaemia[10,11], chronic kidney disease[12], hypertension[13], dyslipidemia[13,14], and macrovascular disease[13]. Referrals to an ophthalmologist are made if retinopathy or other sight threatening pathology is detected, as per Australian National Health and Medical Research Council (NHMRC) guidelines[15]. Referrals are also made to the allied health personnel for immediate assessment and follow-up as required.

To ensure timely and comprehensive communication between the ICDMS and the referring GP, a summary of the assessment and management plan is forwarded to the GP within one week of the patient attending the clinic. This summary also includes any specific recommendations for the GP to follow-up, and informs the GP if the patient has been discharged from the ICDMS. GPs are provided with a direct contact number at the ICDMS for queries, concerns and rapid re-assessment of patients.

### Review and discharge

Phase Three focuses on execution of the management plan developed at the clinic, with the aim of discharging the patient back to their referring GP as soon as targets have been reached and stabilised. Patients newly commenced on insulin, or those who need their insulin regimens revised to achieve optimal glycaemic control are enrolled into the ICDMS insulin stabilisation service (ISS). Patients on the ISS are contacted two or three times a week by a diabetes nurse educator to review their daily blood sugar levels (BSLs). Under supervision of a GP Clinical Fellow, the diabetes nurse educator makes small, incremental adjustments to the patient's insulin regimen to achieve stable and optimal BSLs. Patients are reviewed at the weekly clinic as required to monitor and adjust other treatments. Where appropriate, patients are strongly encouraged to attend either the self-management or weight loss programs provided by the ICDMS or other relevant programs provided by the Inala Community Health Centre.

## Methods/Design

### Study aim and design

**Figure 2:**
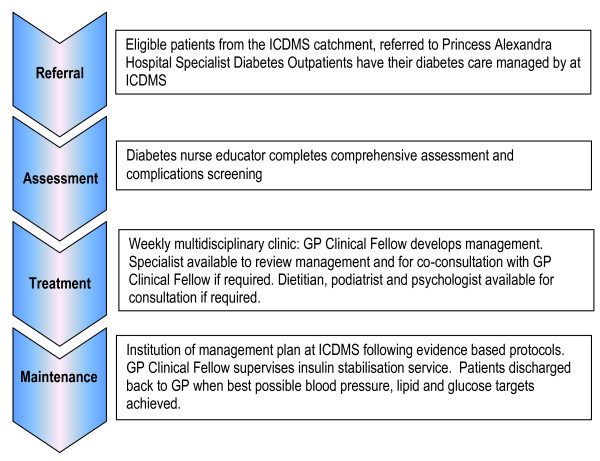
**Inala Chronic Disease Management Service clinical model of care**.

The overarching aim of the ICDMS clinical model is to provide comprehensive and integrated clinical care for people with complex T2DM to achieve optimal glycaemic control. An open controlled intervention trial is comparing the impact of the ICDMS clinical model on patient outcomes compared to usual care provided to comparable patients attending the Princess Alexandra Hospital (PAH) Specialist Diabetes Outpatient Clinic. Figure 2 shows the study design. Usual care at the PAH involves assessment and review by a consultant endocrinologist or supervised training registrars or residents and referral to a diabetes nurse educator or other allied health personnel as required. Patients that have relatively stable control tend to not be discharged back to their referring GP, but continue to attend the clinic for annual reviews.

### Patient population and recruitment

The target population for the ICDMS evaluation study was patients with T2DM who had been referred by their GP to the PAH specialist diabetes outpatient clinic. Both new and existing patients of the specialist diabetes outpatient clinic who were at least 18 years old and able and willing to provide informed consent were eligible to participate. Interpreter services were available for patients who needed assistance with their English. Exclusion criteria included being on haemodialysis, renal transplant patients because of their need for frequent visits to the tertiary hospital, hypoglycaemic unawareness, or pregnancy.

Recruitment of patients to the ICDMS occurred between September 2007 and April 2008. The ICDMS focussed on patients who lived within the Queensland Health designated catchment area for the Inala Community Health Centre or attended a GP who practiced within this area who had been referred by their GP to the PAH specialist outpatient clinic. These patients were identified from the PAH specialist outpatient clinic's appointment booking system. Approval was sought from the patient's GP for the ICDMS to contact the patient by letter and/or phone to invite them to attend the ICDMS rather than the PAH specialist outpatient diabetes clinic. No GP refused consent for their patient to attend the ICDMS, and all patients invited to attend the ICDMS did so. All patients attending the ICDMS underwent the initial comprehensive assessment and complications screening described above, irrespective of previous attendances at the PAH.

One hundred and forty nine consecutive patients with T2DM attending the PAH specialist diabetes outpatients clinic from January to April 2008 were invited to participate in this study as the control group. Their recruitment occurred in the clinic whilst they waited for their appointment with the specialist. Only four patients refused to participate.

### Sample size and power calculation

A total of 240 patients (120 per group) were needed to detect a difference between study groups of 0.5% in the change in HbA1c from baseline, assuming a standard deviation of HbA1c (our primary outcome) of 1.4%, using a 2-sided test with a significance level of 0.05 and 80% power. We estimated a 20% discontinuation rate for the 12 month follow-up period, thus we aimed to enrol 300 patients (150 per group).

### Outcome assessment

Data from patients were collected at baseline by reviewing medical records for clinical data and self-completed questionnaires for demographic data, including age, education level, ethnicity, duration of diabetes, and history of co-morbidities. Clinical data will be collected again at six and 12 months in similar fashions.

The ICDMS aimed to enable patients to achieve optimal glycaemic control and therefore, the primary outcome is HbA1c at 12 months. The proportion of patients with HbA1c ≤ 7% (ie. within the NHMRC guidelines for glycaemic control[15]; between 7.1% and ≤ 8%; between 8.1% and 10%; and greater than 10% will be calculated and compared between groups.

Secondary outcome measures include improvements in modifiable cardiovascular disease risk factors, and improvements in patient quality of life and diabetes self-efficacy. Improvements in modifiable cardiovascular disease risk factors include decreased total and low density lipoprotein (LDL) cholesterol and blood pressure (BP), increased levels of physical activity, and increased proportion of non-smoking participants. Additionally, the proportion of participants with BP and LDL cholesterol at target will be assessed at 12 months. Quality of care indicators included the proportion of patients with microalbuminuria on Angiotensin-Converting Enzyme (ACE) inhibitors or Angiotensin 2 Receptor Antagonists (A2RB); the proportion of patients with dyslipideamia on statins or fibrates (if indicated and tolerated); and the proportion of patients on prophylactic aspirin (as was accepted best practice at the commencement of this study).

Changes in health status will be assessed using the EuroQoL 5-Dimension (EQ-5D) scale. This standardised, five item descriptive system and visual analog scale measures mobility, self-care, pain, usual activities and anxiety and contributes to a 'utility' score[16] and was chosen to enable an international comparison of the cost-effectiveness of the project. Changes in diabetes self efficacy, or an "individual's judgement of confidence to carry out tasks specific to diabetes management" will be assessed using the 18-item Diabetes Self Efficacy Scale. This scale assesses an individual's sense of certainty or uncertainty about their ability to manage their condition, and their perceived ability to follow diabetic routines, self-treat, exercise and follow an appropriate dietary plan[17]. Changes in the prevalence and severity of depression will be assessed in the intervention group using the depression module of Patient Health Questionnaire (PHQ-9)[18] but not in the control group as it was beyond the scope of this project to provide clinical care for any control patients identified with significant levels of depression via this assessment.

### Statistical analysis

Baseline characteristics that differ significantly between the two patient groups and the two groups of general practices will be identified using descriptive statistics and adjusted for in all multivariate analyses. Characteristics of participants who complete and withdraw will be compared. To ensure the generalisability of the results, the number of non-participants, their age and gender, and their reason for non-participation will be recorded.

Analyses of primary outcomes will be done on an intention to treat basis assuming return to baseline values for any patients who do not complete follow-up. Measures of the outcome value at baseline will be included in the regression analysis as a covariable. Other covariables may include any characteristics that are not balanced between the groups at baseline. A sensitivity analysis will investigate the effect of participant loss to follow-up on the intervention effect. Statistical significance will be based on two-tailed tests, with p < 0.05 considered significant.

### Baseline results

A total of 335 patients participated in this study: 185 in the ICDMS group and 145 in the control group. Only four patients in the control group declined to participate in the study.

Baseline clinical characteristics and demographic data for control and intervention patients are presented in Table 1. There were no significant differences between groups for known duration of diabetes, self-reported diabetes treatment, country of origin, smoking status, level of physical activity, Body Mass Index (BMI), weight and serum cholesterol. ICDMS patients were significantly younger, were less likely to be male, had significantly lower levels of education, higher levels of HbA1c, lower blood pressure, and were less likely to have neuropathy.

**Table 1:** Demographic and clinical characteristics of participants in usual care (n = 145) and ICDMS (n = 185) groups.

	Usual Care		ICDMS		P
Age in years, mean (standard deviation (SD))	62.9	(11.6)	59.4	(13.4)	0.01

Duration of diagnosed diabetes in years, mean (SD)	13.7	(0.9)	12.8	(0.8)	0.47

Diabetes treatment					0.68

*Non-pharmacological*	5	(4%)	5	(3%)	

*Oral hyperglycaemics*	61	(46%)	68	(41%)	

*Oral hyperglycaemics and insulin*	41	(31%)	62	(37%)	

*Insulin only*	25	(19%)	32	(19%)	

Sex (male)	93	(64%)	83	(45%)	0.001

Country of origin					0.30

*Australia*	87	(61%)	78	(51%)	

*UK and Ireland*	13	(9%)	17	(11%)	

*New Zealand*	9	(6%)	7	(5%)	

*Europe*	12	(8%)	12	(8%)	

*Asia*	9	(6%)	13	(9%)	

*Other*	13	(9%)	26	(17%)	

Aboriginal or Torres Strait Islander	3	(2%)	8	(4%)	0.36

Smoking					0.74

*Never*	55	(42%)	64	(39%)	

*Ex-smoker*	59	(45%)	74	(45%)	

*Current smoker*	17	(13%)	26	(16%)	

Education level					0.51

*No formal qualifications*	36	(27%)	36	(23%)	

*School/Intermediate Certificate*	25	(19%)	34	(21%)	

*Higher School Certificate*	25	(19%)	40	(25%)	

*Diploma or Apprenticeship*	35	(27%)	42	(26%)	

*University Degree*	11	(8%)	8	(5%)	

BMI (kg/m^2^), mean (SD)	31.8	(6.3)	33.3	(8.3)	0.08

Weight (kg), mean (SD)	91.5	(22.5)	91.0	(25.9)	0.85

HbA1_c _(%), mean (SD)	7.9	(1.9)	8.5	(1.9)	0.001

HbA1_c _≤ 7%	54	(38%)	37	(22%)	0.003

Sitting systolic BP (mmHg), mean (SD)	140	(19)	130	(16)	<0.001

Sitting diastolic BP (mmHg), mean (SD)	77	(11)	72	(11)	<0.001

Blood Pressure ≤ 130/80 mmHg, mean	48	(33%)	99	(56%)	<0.001

Total Cholesterol (mmol/L), mean (SD)	4.2	(1.1)	4.3	(1.1)	0.47

HDL-cholesterol (mmol/L), mean (SD)	1.1	(0.3)	1.1	(0.4)	0.69

LDL-cholesterol (mmol/L), mean (SD)	2.5	(2.4)	2.4	(0.8)	0.50

LDL-cholesterol ≤ 2.5 (mmol/L)	86	(67%)	96	(63%)	0.62

Triglycerides, mean (SD)	1.9	(1.5)	2.3	(4.1)	0.37

Neuropathy	73	(56%)	53	(31%)	<0.001

Retinopathy	53	(41%)	36	(33%)	0.28

Ischaemic heart disease	38	(34%)	56	(39%)	0.51

Variables in usual care group with missing data: 1 observation missing for HbA1_c_; 3 observations missing for triglycerides; 5 observations missing for Aboriginality; 11 observations missing for duration of diabetes and HDL-cholesterol; 13 observations missing for diabetes treatment, country of origin, and highest education level; 14 observations missing for smoking and retinopathy; 15 observations missing for neuropathy; 16 observations missing for LDL-cholesterol; and 33 observations missing for ischaemic heart disease.

Variables in ICDMS group with missing data: 5 observations missing for Aboriginality; 8 observations missing for weight and BMI; 9 observations missing for blood pressure; 12 observations missing for neuropathy; 14 observations missing for total cholesterol; 15 observations missing for triglycerides; 16 observations missing for HbA1_c_; 18 observations missing for diabetes treatment; 21 observations missing for country of origin and smoking status; 25 observations missing for highest education level; 29 observations missing for HDL-cholesterol; 33 observations missing for LDL-cholesterol; 40 observations missing for ischaemic heart disease; 41 observations missing for duration of diabetes; and 76 observations missing for retinopathy.

### Trial organisation and management

The ICDMS received ethical approval from the PAH Human Research Ethics Committee: 2007/100. No significant risks to patients or GPs were anticipated, and as the study was unblinded and low risk a data monitoring committee was not necessary. The trial is registered with the Australian New Zealand Clinical Trials Registry, ACTRN12608000010392. http://www.anzctr.org.au/default.aspx

A steering group comprising of representatives of Queensland Health, The University of Queensland, and the local Division of General Practice provided strategic oversight to the project. In addition, operational and evaluation committees provided input into the day-to-day management of the project.

## Discussion

The increasing prevalence of T2DM in the community is placing significant demands on health care systems. The pivotal role of primary care in providing care for people with T2DM is increasingly being recognised, as is the need to develop new models of care that deliver improved quality, efficiency and patient engagement in care.

We believe the ICDMS is a unique model of diabetes care. It is not a specialist outreach service where a specialist health service is provided to a community on a visiting basis. It is a new model of care delivered by GPs who have undertaken advanced skills training (the ICDMS GP Clinical Fellows) with on-site specialist support from the endocrinologist and a multidisciplinary team. The initial comprehensive assessment ensures efficiency of the consultation between the patient and the GP Clinical Fellow. The support provided by the endocrinologist to GP Clinical Fellows not only ensures the quality of care provided to the patients, but also contributed to the continuing development of the GP Clinical Fellows' knowledge and skills of complex diabetes management.

There are some similarities to specialist primary care diabetes clinics seen in the United Kingdom. Similar to the UK model of GPs with Special Interests (GPwSI), care is provided by GPs who have undertaken additional training in a specific clinical area. The UK GPwSIs take referrals for the assessment or treatment of patients that may otherwise be referred directly to a secondary care consultant: in other words, the GPs practice within the traditional model of the separation of primary and secondary care[19]. In comparison, the ICDMS clinical model features the GP Clinical Fellow, the on-site endocrinologist, the diabetes nurse educator, and the allied health personnel working together to provide enhanced care for patients in a primary care setting.

The ICDMS is also not a general practice diabetes clinic where a general practice will have a clinical session devoted to the care of its patients with T2DM. Patients attending the ICDMS have been referred by their GP for specialist level care which is provided by the ICDMS multidisciplinary team. Once glycaemic control is achieved and the acute complications have been addressed, the patient is discharged back to their referring GP. The ICDMS does not provide a general practice service, and other health issues not related to diabetes are not addressed during the consultations, and patients are advised to discuss these issues with their own GP.

This study does have limitations. We have used a geographical control to assess the effectiveness of the ICDMS clinical model, as this was the most feasible study design in the health service environment in which the ICDMS was established. The differences at baseline between the intervention and control groups demonstrate the difficulties associated with this design, and the need to account for these differences in the statistical analysis. This research was conducted contemporaneously with the delivery of the clinical service. The majority of clinicians involved in the service were not researchers and were principally focused on clinical care rather than ensuring the integrity of the experimental design. At times this was challenging for the clinicians as they struggled with the relative rigidity of implementing a research protocol compared with clinical service delivery that can be adjusted as deemed appropriate by individual clinicians. Replication of this model of care requires a greater understanding of the impact of this model on the providers of care: their roles, satisfaction, and self-efficacy to provide treatment could be investigated through focus groups in both settings. However, this study focused on the impact of the model of care on clinical outcomes, rather than providers. Further research is needed to investigate the later important area.

We believe that the ICDMS clinical model has considerable potential, and could be transferable to other chronic conditions and other geographical areas. Its strengths include the comprehensive assessment and complications screening process, the GP Clinical Fellows value-adding to the clinical efficiency of the specialist thereby increasing patient throughput, and its location within the patients' community. We also believe that the ICDMS clinical model combines the best of two health care domains - the patient focus and holistic care valued by the primary care sector with the specialised knowledge and skills of hospital diabetes care. Our study will provide empirical evidence about the clinical effectiveness and the economic benefits of this model of care.

## Competing interests

The authors declare that they have no competing interests.

## Authors' contributions

DA participated in the design of the study and drafted the manuscript. CJ and AR contributed to the design of the clinical model and the evaluation study. AR also participated in the coordination of the study. RW contributed to the design of the study and performed the statistical analysis. All authors read and approved the final manuscript.

## Pre-publication history

The pre-publication history for this paper can be accessed here:

http://www.biomedcentral.com/1472-6963/10/134/prepub

## References

[B1] Wild S Roglic G Green A Sicree R King H Global prevalence of diabetes: estimates for the year 2000 and projections for 2030 Diabetes Care 2004 27 1047 53 10.2337/diacare.27.5.1047 15111519

[B2] Australian Institute of Health and Welfare Diabetes: Australian facts 2008 Diabetes series: no. 8; Cat. no. CVD 40 2008 Canberra: AIHW

[B3] Queensland Health The Health of Queenslanders 2008: Prevention of Chronic Disease 2008 Second Repot of the Chief Health Officer Queensland. Brisbane: Queensland Health

[B4] Stratton IM Cull CA Adler AI Matthews DR Neil HA Holman RR Additive effects of glycaemia and blood pressure exposure on risk of complications in type 2 diabetes: a prospective observational study (UKPDS 75) Diabetologia 2006 49 1761 9 10.1007/s00125-006-0297-1 16736131

[B5] Diabetes Australia Diabetes Management in General Practice 2008/09: Guidelines for Type 2 Diabetes 2008 14 Canberra: Diabetes Australia

[B6] Britt H Miller G Charles J Bayram C Pan Y Henderson J Valenti L O'Halloran J Harrison C Fahridin S General practice activity in Australia 2006-07 2008 General practice series no. 21. Cat. no. GEP 21. General Practice. Canberra: Australian Institute of Health and Welfare

[B7] Bodenheimer T Lorig K Holman H Grumbach K Patient self-management of chronic disease in primary care JAMA 2002 288 2469 75 10.1001/jama.288.19.2469 12435261

[B8] Zwar N Harris M Griffiths R Roland M Dennis S Powell Davies G Hasan I A systematic review of chronic disease management 2006 Sydney: Research Centre for Primary Health Care and Equity, School of Public Health and Community Medicine. UNSW

[B9] Askew D Schluter PJ Spurling G Maher CM Cranstoun P Kennedy C Jackson C Diabetic retinopathy screening in general practice: a pilot study Aust Fam Physician 2009 38 650 6 19893789

[B10] Nathan DM Buse JB Davidson MB Ferrannini E Holman RR Sherwin R Zinman B Management of hyperglycemia in type 2 diabetes: a consensus algorithm for the initiation and adjustment of therapy: update regarding thiazolidinediones: a consensus statement from the American Diabetes Association and the European Association for the Study of Diabetes Diabetes Care 2008 31 173 5 10.2337/dc08-9016 18165348

[B11] Nathan DM Buse JB Davidson MB Heine RJ Holman RR Sherwin R Zinman B Management of hyperglycemia in type 2 diabetes: A consensus algorithm for the initiation and adjustment of therapy: a consensus statement from the American Diabetes Association and the European Association for the Study of Diabetes Diabetes Care 2006 29 1963 72 10.2337/dc06-9912 16873813

[B12] Chronic Kidney Disease (CKD) Management in General Practice 2007 Melbourne: Kidney Health Australia

[B13] Australian Centre for Diabetes Strategies National Evidence Based Guidelines for the Management of Type 2 Diabetes Mellitus: Prevention and Detection of Macrovascular Disease in Type 2 Diabetes 2004 Canberra: National Health and Medical Research Council

[B14] Tonkin A Barter P Best J Boyden A Furler J Hossack K Sullivan D Thompson P Vale M Cooper C Robinson M Clune E National Heart Foundation of Australia and the Cardiac Society of Australia and New Zealand: position statement on lipid management--2005 Heart Lung Circ 2005 14 275 91 10.1016/j.hlc.2005.10.009 16361000

[B15] Australian Diabetes Society Guidelines for the Management of Diabetic Retinopathy 2008 Canberra: National Health and Medical Research Council

[B16] EuroQol--a new facility for the measurement of health-related quality of life. The EuroQol Group Health Policy 1990 16 199 208 10.1016/0168-8510(90)90421-9 10109801

[B17] Rapley P Passmore A Phillips M Review of the psychometric properties of the Diabetes Self-Efficacy Scale: Australian longitudinal study Nurs Health Sci 2003 5 289 97 10.1046/j.1442-2018.2003.00162.x 14622381

[B18] Kroenke K Spitzer RL Williams JB The PHQ-9: validity of a brief depression severity measure J Gen Intern Med 2001 16 606 13 10.1046/j.1525-1497.2001.016009606.x 11556941 PMC1495268

[B19] Royal College of General Practitioners General Practitioners with a Special Interest (GPwSI) 2009 London: Royal College of General Practitioners

